# Association between three genetic variants in kallikrein 3 and prostate cancer risk

**DOI:** 10.1042/BSR20181151

**Published:** 2018-11-30

**Authors:** Wei-Hong Ding, Ke-Wei Ren, Chuang Yue, Jian-Gang Zou, Li Zuo, Li-Feng Zhang, Yu Bai, Atsushi Okada, Takahiro Yasui, Yuan-Yuan Mi

**Affiliations:** 1 Department of Urology, Huashan Hospital, Fudan University, 12 Central Urumqi Road, Shanghai 200040, China; 2 Department of Orthopedics, the Affiliated Jiangyin Hospital of Southeast University Medical School, Jiangyin 214400, China; 3 Department of Urology, The Affiliated Changzhou No. 2 People’s Hospital of Nanjing Medical University, Changzhou 213003, Jiangsu Province, China; 4 Department of Nephrourology, Nagoya City University Graduate School of Medical Sciences, Aichi 4678601, Japan; 5 Department of Urology, Third Affiliated Hospital of Nantong University (Affiliated Hospital of Jiangnan University), 585 Xingyuan Road, Wuxi 214041, China

**Keywords:** Analysis, ELISA, KLK3, Polymorphism, Prostate cancer

## Abstract

Background: Epidemiological studies have assessed the association between kallikrein 3 (KLK3) polymorphisms and prostate cancer (PCa) susceptibility. However, published data on this association are somewhat inconclusive. Methods: Articles investigating the association between three KLK3 (rs1058205, rs2735839, and rs266882) variants and PCa susceptibility were searched from online databases, which included 35,838 patients and 36,369 control participants. Odds ratios (ORs) with 95% confidence intervals (CIs) were used to demonstrate the strength of the association. We also utilized ELISA to detect serum expression of KLK3. In addition, *in silico* tools were adopted to evaluate the relationship of KLK3 expression and PCa survival time. Results: The overall results indicated that polymorphism T>C of rs1058205 was associated with decreased risk of PCa (allele contrast: OR = 0.75, 95% CI = 0.64–0.88, *P*_heterogeneity_ < 0.001; homozygote comparison: OR = 0.58, 95% CI = 0.42–0.81, *P*_heterogeneity_ < 0.001), particularly in Caucasian population (allele contrast: OR = 0.77, 95% CI = 0.65–0.91, *P*_heterogeneity_ < 0.001; homozygote comparison: OR = 0.58, 95% CI = 0.41–0.82, *P*_heterogeneity_ < 0.001). No association was observed between the polymorphism A>G of rs2735839 and risk of PCa. In addition, no association was observed between polymorphism A>G of rs266882 and risk of PCa. Serum KLK3 levels in PCa patients carrying CC/CT genotypes were statistically lower than those carrying TT genotypes. Conclusion**:** This meta-analysis suggests that rs1058205 polymorphism of KLK3 is a risk factor for PCa development, polymorphism T>C of rs1058205 is associated with decreased susceptibility to PCa particularly in Caucasian population.

## Introduction

Prostate cancer (PCa) is one of the most common cancers among men in U.S.A. [1]. However, the exact etiology underlying the development and progression of PCa is still poorly understood. Previous studies indicated that both environmental and genetic factors may be associated with the development and progression of PCa [2,3]. Single nucleotide polymorphism (SNP) is considered as the most common type of genetic variation and has been reported to be related to increased susceptibility to PCa [4,5]. In recent decades, genome-wide association studies (GWAS) in multiple ethnicities have identified some susceptibility genes in PCa. However, the molecular mechanisms of these SNPs remain poorly defined [6].

Serum levels of prostate-specific antigen (PSA) are measured to screen for PCa. Approximately 40–45% of variations in PSA serum levels are estimated to be influenced by genetic components [7,8]. A previous GWAS on European population identified six genetic loci that were significantly associated with PSA level [9]. Although genetic analyses of PSA levels from Asian countries are relatively limited compared with the European countries, GWAS from China has demonstrated that three loci, solute carrier family 45 member 3 (SLC45A3), microseminoprotein-β (MSMB) and kallikrein 3 (KLK3), were significantly associated with PSA levels [10]. The KLK locus is clustered in a tandem array of approximately 300 kb on chromosome 19q13.4 and contains the largest cluster of 15 homologous protease genes. In addition, the KLK3 gene is a serine protease kallikrein family member and may be involved in the occurrence and metastasis of PCa [11,12].

A number of epidemiological studies have explored the relationship between KLK3 polymorphisms and PCa risk. Previous studies have shown that the TT genotype of rs1058205 was associated with decreased PCa aggressiveness in the Caucasian population based on the Gleason score [13]. However, Zhang and colleagues found no correlation between any of the SNPs at the 19q13.33 locus and PCa risk in Han Chinese men [14]. Various case–control studies showed inconsistent results on the association between KLK3 polymorphism and PCa. Hence, in our meta-analysis, accumulated data from all eligible studies were utilized to summarize and enhance the statistical powers of these published studies for the three KLK3 variants. All the articles that met the selection criteria were included in the present analysis. The pooled results from all these studies allowed us to draw novel conclusions.

## Materials and methods

### Search strategy and identification of eligible studies

To identify all potentially eligible studies on KLK3 polymorphisms and PCa risk, a systematic search was carried out on PubMed, Embase and SinoMed (from China) databases, covering all researches published literature worldwide due to July 30, 2017. The search terms “kallikrein 3 or KLK3,” “polymorphism or variant,” and “prostate cancer or tumor or adenocarcinoma” were used to identify suitable literature. Furthermore, review articles and bibliographies of other studies were identified through a manual search. All full-text case–control studies were included if they had PCa risk and KLK3 polymorphism, sufficient available data to estimate an odds ratio (OR) with 95% confidence interval (CI) and the frequencies of the allele or genotype available.

### Data extraction and quality assessment

Two researchers independently extracted and reviewed the information complying with the selection criteria. In the case of a disagreement, the researchers discussed the studies until a consensus was reached. For each study, the following characteristics were summarized: first author’s last name, year of publication, sample size of cases and controls, country of origin, ethnicity, study-design (sources of samples), age range, the number of cases and controls with variant allele and wild type, and genotyping methods. Finally, nine articles were included [15–23] and the Newcastle–Ottawa scale (NOS) score was shown in Table 1.

**Table 1:** Basic information for included studies of the association between three KLK3 polymorphisms and prostate cancer susceptibility

Author [Ref.]	Year	Origin	Ethnicity	Source	Case	Control	Case			Control			HWE	Age range		Method	NOS
														**Case**	**Control**		
**rs2735839**							**GG**	**GA**	**AA**	**GG**	**GA**	**AA**					
Hu [15]	2014	China	Asian	PB	108	242	25	68	15	44	108	90	0.25	70.01 ± 7.26	70.52 ± 7.11	TaqMan	8
Wang [19]	2013	China	Asian	PB	285	280	102	126	57	98	144	38	0.19	NA	NA	HRM-PCR	7
Eeles [18]	2008	U.K.	Caucasian	PB	1851	1887	35	406	1410	103	591	1193	0.01	NA	NA	Illumina array	7
Parikh [22]	2011	U.S.A.	Caucasian	PB	3150	2980	260	299	2591	261	288	2431	<0.01	NA	NA	GWAS	8
**rs266882**							**GG**	**GA**	**AA**	**GG**	**GA**	**AA**					
Cicek [16]	2005	U.S.A.	Caucasian	FB	439	479	119	196	124	119	236	124	0.75	NA	NA	PCR-RFLP	6
Lai [17]	2007	Australia	Caucasian	PB	209	223	35	111	63	71	107	45	0.68	68 (50–91)	50 (18–75)	PCR-RFLP	8
Penney [20]	2011	U.S.A.	Caucasian	PB	966	1279	255	483	228	346	622	311	0.34	59 ± 8.3	59.3 ± 8.0	Sequenom technology	8
**rs1058205**							**CC**	**CT**	**TT**	**CC**	**CT**	**TT**					
Penney [20]	2011	U.S.A.	Caucasian	PB	896	1165	14	176	706	28	269	868	0.19	59 ± 8.3	59.3 ± 8.0	Sequenom technology	8
Chen [21]	2017	China	Asian	HB	268	298	4	55	209	7	89	202	0.44	58 (52–62)	62 (52–67)	HRM-PCR	7
Eeles [18]	2008	U.K.	Caucasian	PB	1850	1887	54	490	1306	136	674	1077	0.03	NA	NA	Illumina array	7
Stegeman [23]	2015	Australia	Caucasian	PB	22301	22320	446	5798	161057	670	6476	15177	0.53	64.8 ± 8.0	60.6 ± 10.7	Illumina array	8
Parikh [22]	2011	U.S.A.	Caucasian	PB	3515	3329	373	329	2813	410	298	2621	<0.01	NA	NA	GWAS	8

Abbreviations: FB, family-based; GWAS, Genome-Wide Association Study; HB, hospital-based; HRM-PCR, high-resolution melting curve polymerase chain reaction; HWE, Hardy–Weinberg equilibrium of controls; NA, not available; NOS, Newcastle–Ottawa scale; PB, population-based; PCR-FLIP, polymerase chain reaction and restrictive fragment length polymorphism.

### Enzyme-linked immunosorbent assay (ELISA)

We collected the blood of participants in standard cubes without anticoagulant according to the instructions. Patients were enrolled from the Affiliated Changzhou No.2 People’s Hospital of Nanjing Medical University and third Affiliated Hospital of Nantong University. Then, we centrifuged the blood sample immediately at 1000 rpm for 20 min by ELISA kit (Thermo Scientific Ltd.). A standard ELISA plate reader was used for measuring absorbance at 450 and 550 nm. We correlated the absorbance against a standard curve. Written informed consent by each participant was obtained before samples were collected. The present study was approved by Institution Review Board of these two hospitals.

### Statistical analysis

The strength of the association between the three SNPs and PCa risk was evaluated by OR with its corresponding 95% CI. The statistical significance of the OR was evaluated with a *Z*-test. The heterogeneity assumption among studies was estimated utilizing a chi-square-based *Q*-test. The wild-type homozygote (WW), the risk of rare allele homozygote (RR), and heterozygous (WR) genotypes were evaluated. Then we assessed the risk of PCa under a dominant model (RR+WR vs. WW) and recessive model (RR vs. WR+WW). Subgroup analysis was stratified by ethnicity and source of control (hospital-based, HB, and population-based, PB). A *P*-value of greater than 0.10 for the *Q*-test indicated a lack of heterogeneity among the studies. If a significant heterogeneity was detected, the DerSimonian–Laird (random-effects model) was applied; otherwise the Mantel–Haenszel (fixed-effects model) was performed to calculate the pooled OR [24,25]. Hardy–Weinberg equilibrium (HWE) was calculated using the Pearson’s chi-square test. Publication bias was assessed using both Egger’s test and Begg’s test, and a *P*-value of less than 0.05 was considered significant [26]. All statistical tests for our meta-analysis were carried out using the Stata software version 11.0 (StataCorp LP, College Station, TX, U.S.A.).

### Quality score assessment, sensitivity analysis, and *in silico* analysis

The NOS was used to evaluate the quality of each study [27]. The NOS is a tool utilized for evaluating the quality of studies included in meta-analysis. Stars are awarded such that the highest quality studies are awarded up to nine stars. In the present analysis, studies with more than seven stars should be considered as high quality. We conducted leave-one-out sensitivity analysis to assess the stability of the overall results. The sensitivity was evaluated by removing each research once at a time in every genetic model for the three KLK3 variants. Furthermore, we used the online gene expression mini database to demonstrate the KLK3 expression in prostate tissues (http://gemini.cancer-pku.cn/).

## Results

### Study characteristics

**Figure 1:**
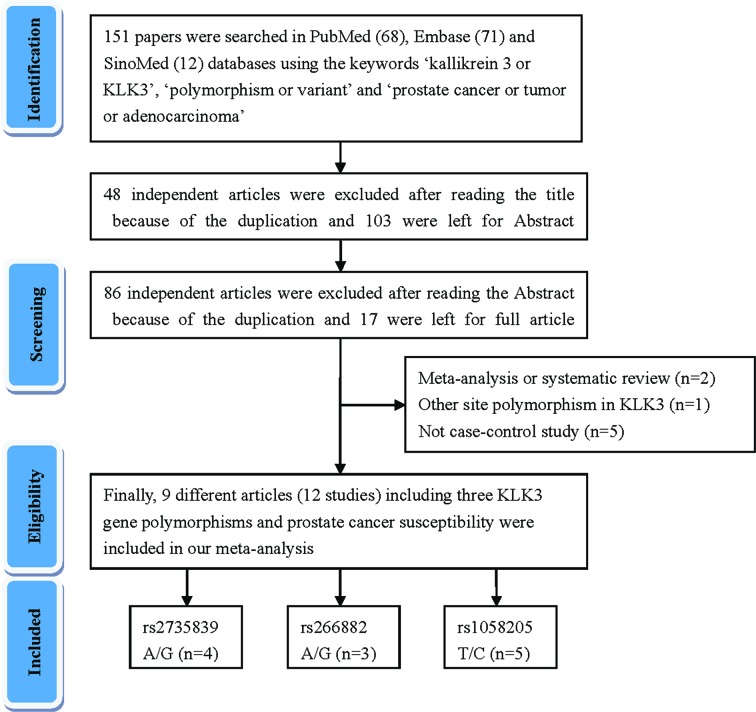
Flowchart illustrating the search strategy used to identify several studies investigating the association between three KLK3 variants and prostate cancer risk

**Figure 2:**
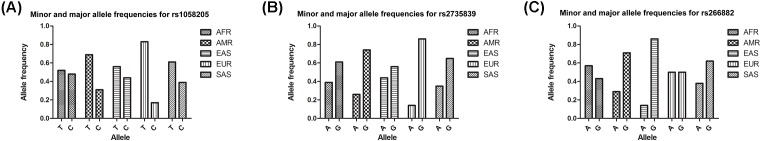
Minor allele and major allele frequencies for KLK3 rs1058205 (**A**), rs2735839 (**B**), and rs266882 (**C**) polymorphisms in the controls stratified by ethnicity. Vertical axis, allele frequency; horizontal axis, allele type. AMR: American; AFR: African; EAS: East Asian; SAS: South Asian; EUR: European.

A total of nine articles (including 12 case–control studies) met the above selection criteria and are included in our meta-analysis (Figure 1). The characteristic of these studies were summarized in Table 1. Furthermore, we checked the minor allele frequency (MAF) of three KLK3 variants reported for the main worldwide populations from 1000 genomes online. The MAFsof rs1058205 were as follows: American (AMR), 0.308; African (AFR), 0.477; East Asian (EAS), 0.442; South Asian (SAS), 0.395; and European (EUR), 0.171. Then, those of rs2735839 were as follows: AMR, 0.264; AFR, 0.391; EAS, 0.442; SAS, 0.347; and EUR, 0.140. At last, those of rs266882 were as follows: AMR, 0.290; AFR, 0.567; EAS, 0.144; SAS, 0.377; and EUR, 0.504 (Figure 2). Overall, 6872 PCa patients and 7740 control participants with three KLK3 polymorphisms were evaluated. In the subgroup of ethnicity, six studies were performed in individuals of European descent and three studies were in Asian descendants. Population-based controls were carried out in seven of the studies. Polymerase chain reaction-restriction fragment length polymorphism (RFLP), the classical genotyping method, was utilized in two of the comparisons. The distribution of genotypes in seven controls was consistent with HWE.

### Quantitative synthesis

**Figure 3:**
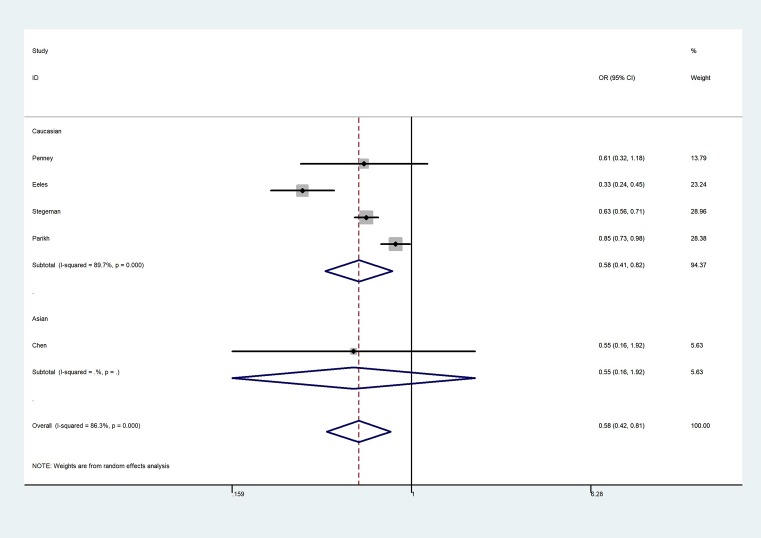
Forest plot of CC vs. TT genetic model of KLK3 rs1058205 polymorphism

**Figure 4:**
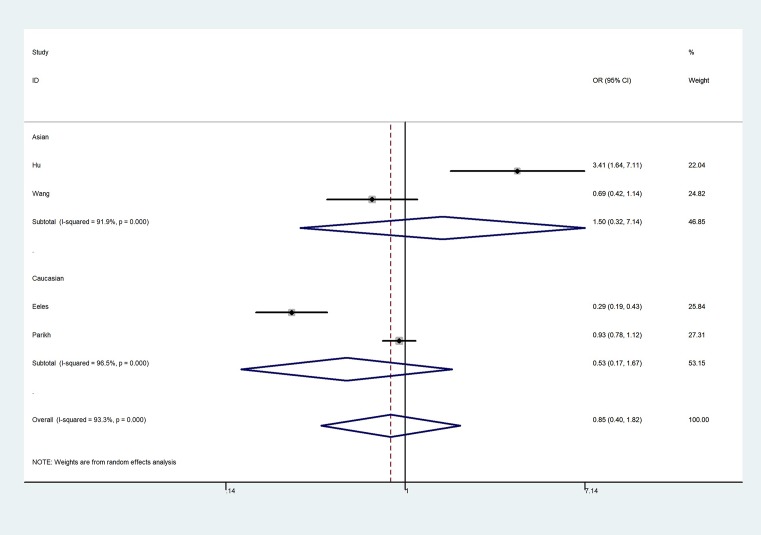
Forest plot of GG vs. AA genetic model of KLK3 rs2735839 polymorphism

**Figure 5:**
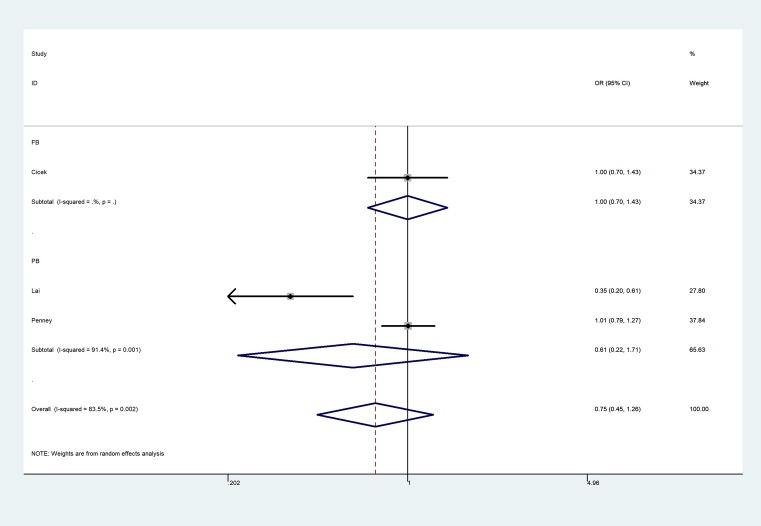
Forest plot of GG vs. AA genetic model of KLK3 rs266882 polymorphism

The overall results indicated a significant association between the rs1058205 T>C polymorphism and PCa (allele contrast: OR = 0.75, 95% CI = 0.64–0.88, *P*_heterogeneity_ < 0.001; heterozygote comparison: OR = 0.787, 95% CI = 0.65–0.93, *P*_heterogeneity_ < 0.001; homozygote comparison: OR = 0.58, 95% CI = 0.42–0.81, *P*_heterogeneity_ < 0.001, Figure 3; dominant genetic model: OR = 0.74, 95% CI = 0.62–0.88, *P*_heterogeneity_ < 0.001; and recessive genetic model: OR = 0.63, 95% CI = 0.48–0.82, *P*_heterogeneity_ < 0.001) (Table 2), especially in the Caucasian population (allele contrast: OR = 0.77, 95% CI = 0.65–0.91, *P*_heterogeneity_ < 0.001; heterozygote comparison: OR = 0.80, 95% CI = 0.66–0.98, *P*_heterogeneity_ < 0.001; homozygote comparison: OR = 0.58, 95% CI = 0.41–0.82, *P*_heterogeneity_ < 0.001; dominant genetic model: OR = 0.76, 95% CI = 0.63–0.92, *P*_heterogeneity_ < 0.001; and recessive genetic model: OR = 0.63, 95% CI = 0.47–0.83, *P*_heterogeneity_ < 0.001. However, no association was observed between the rs2735839 A>G polymorphism and PCa risk in allele contrast (OR = 0.93, 95% CI = 0.63–1.39, *P*_heterogeneity_ < 0.001), heterozygote comparison (OR = 0.98, 95% CI = 0.59–1.64, *P*_heterogeneity_ < 0.001); homozygote comparison (OR = 0.85, 95% CI = 0.40–1.82, *P*_heterogeneity_ < 0.001, Figure 4); dominant genetic model (OR = 0.97, 95% CI = 0.58–1.61, *P*_heterogeneity_ < 0.001); and recessive genetic model (OR = 0.81, 95% CI = 0.48–1.34, *P*_heterogeneity_ < 0.001). In addition, no association was observed between rs266882 A>G variant and PCa risk in allele contrast (OR = 0.87, 95% CI = 0.67–1.12, *P*_heterogeneity_ = 0.003); heterozygote comparison (OR = 0.95, 95% CI = 0.81–1.12, *P*_heterogeneity_ = 0.240); homozygote comparison (OR = 0.75, 95% CI = 0.45–1.26, *P*_heterogeneity_ = 0.002, Figure 5); dominant genetic model (OR = 0.86, 95% CI = 0.65–1.15, *P*_heterogeneity_ = 0.060); and recessive genetic model (OR = 0.66, 95% CI = 0.30–1.46, *P*_heterogeneity_ = 0.001).

**Table 2:** Stratified analyses of three KLK3 polymorphisms on prostate cancer risk

			R-allele vs. W-allele			WR vs. WW			RR vs. WW			RR+WR vs. WW			RR vs. WR+WW		
Variables	*N*	Case/Control	OR (95%CI)	*P*_heter_*	*P*	OR (95%CI)	*P*_heter_*	*P*	OR (95%CI)	*P*_heter_*	*P*	OR (95%CI)	*P*_heter_*	*P*	OR (95%CI)	*P*_heter_*	*P*
rs2735839	4	5394/5389	0.93 (0.63–1.39)	<0.001	0.736	0.98 (0.59–1.64)	<0.001	0.947	0.85 (0.40–1.82)	<0.001	0.678	0.97 (0.58–1.61)	<0.001	0.906	0.81 (0.48–1.34)	<0.001	0.407
Ethnicity																	
Asian	2	393/522	1.24 (0.63–2.44)	0.001	0.526	1.47 (0.23–9.20)	<0.001	0.682	1.50 (0.32–7.14)	<0.001	0.609	1.50 (0.26–8.56)	<0.001	0.647	1.11 (0.83–1.49)	0.418	0.469
Caucasian	2	5001/4867	0.72 (0.42–1.24)	<0.001	0.236	0.75 (0.45–1.25)	<0.001	0.267	0.53 (0.17–1.67)	<0.001	0.276	0.72 (0.41–1.26)	<0.001	0.248	0.57 (0.21–1.56)	<0.001	0.274
rs266882	3	1614/1981	0.87 (0.67–1.12)	0.003	0.276	0.95 (0.81–1.12)	0.240	0.533	0.75 (0.45–1.26)	0.002	0.274	0.86 (0.65–1.15)	0.062	0.305	0.81 (0.53–1.25)	0.002	0.347
Source of control																	
PB	2	1175/1502	0.79 (0.48–1.29)	0.001	0.349	1.00 (0.83–1.21)	0.170	0.981	0.61 (0.22–1.71)	0.001	0.352	0.81 (0.46–1.41)	0.060	0.456	0.66 (0.30–1.46)	0.001	0.310
rs1058205	5	28830/28999	0.75 (0.64–0.88)	<0.001	<0.001	0.78 (0.65–0.93)	<0.001	0.007	0.58 (0.42–0.81)	<0.001	0.001	0.74 (0.62–0.88)	<0.001	0.001	0.63 (0.48–0.82)	<0.001	0.001
Ethnicity/source of control																	
Caucasian/PB	4	28562/28701	0.77 (0.65–0.91)	<0.001	0.002	0.80 (0.66–0.98)	<0.001	0.027	0.58 (0.41-0.82)	<0.001	0.002	0.76 (0.63–0.92)	<0.001	0.005	0.63 (0.47–0.83)	<0.001	0.001

*P value of Q-test for heterogeneity test (*P*_heter_)

### Serum expression of KLK3, sensitivity analysis, and *in silico* analysis

**Figure 6:**
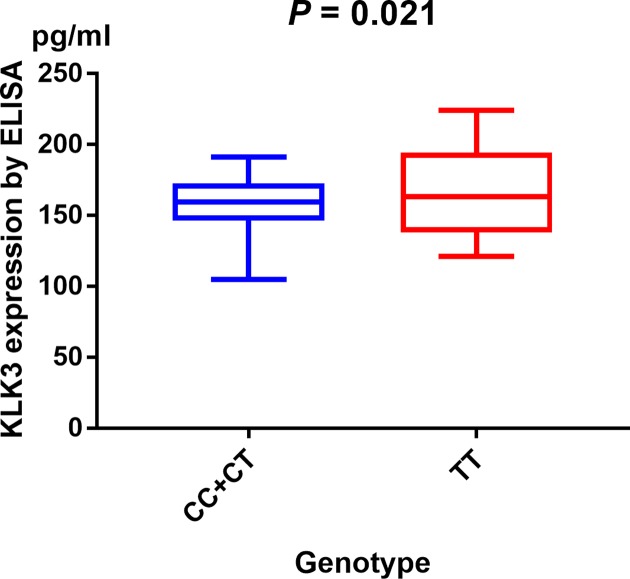
Analysis of serum KLK3 levels in rs1058205 T>C genotype of PCa cases with mean values by ELISA (horizontal lines, mean values)

**Figure 7:**
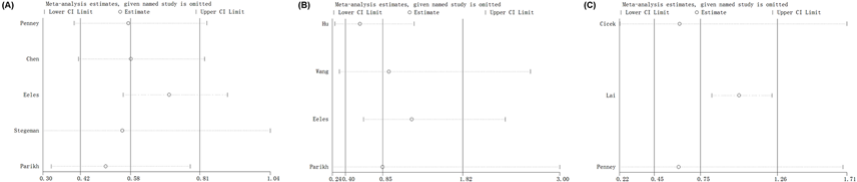
Sensitivity analysis about KLK3 rs1058205 (**A**), rs2735839 (**B**), and rs266882 (**C**) polymorphisms and PCa risk

**Figure 8:**
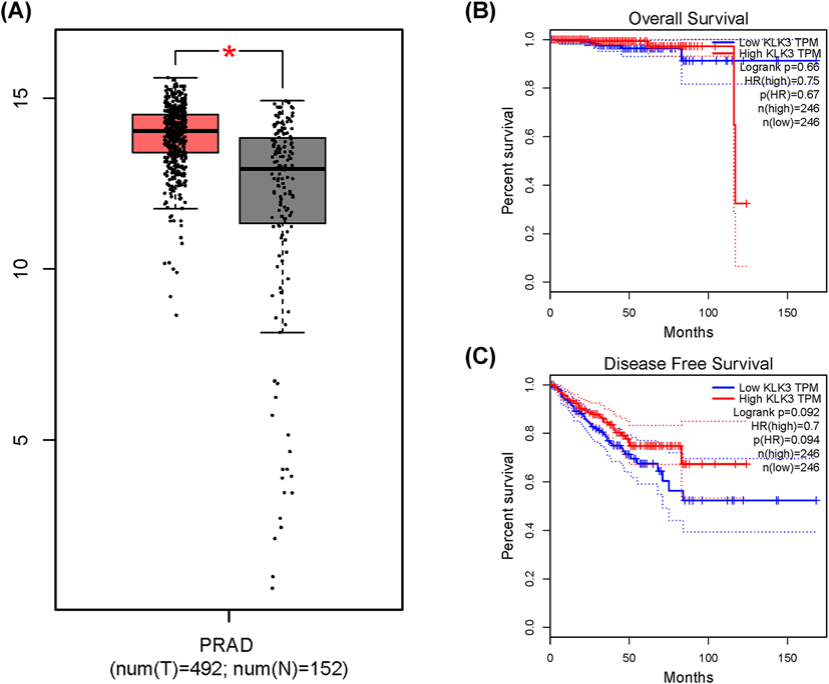
*In silico* analysis of KLK3 expression (**A**) Relative expression of KLK3 in PCa tissue and paracancerous tissue. The association between KLK3 expression and overall survival time (**B**) or disease free survival time (**C**) in prostate cancer patients.

Serum expression of KLK3 was investigated by ELISA. Our research show evidence that serum KLK3 levels in PCa participants with CC/CT genotypes were relatively lower than those with TT genotypes (*P* = 0.021, Figure 6). We measured sensitivity by removing each study once a time in every genetic model for KLK3 rs1058205, rs2735839, and rs266882 variants. Results of sensitivity analysis indicated that no individual study could influence the pooled ORs (Figure 7). *In silico* analysis showed that KLK3 expression in PCa tissue was relatively higher than that in control tissue (Figure 8A). Furthermore, we assessed the correlation between KLK3 expression and overall survival (OS) or disease-free survival (DFS) time of PCa patients. Kaplan–Meier estimate identified no significant difference for either OS time (*P* = 0.67, Figure 8B) or DFS time (*P* = 0.094, Figure 8C).

### Publication bias

**Figure 9:**
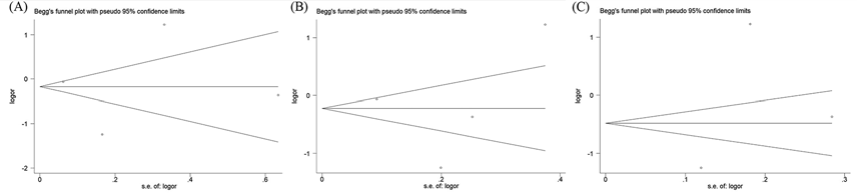
Begg’s funnel plot of standard error for assessing publication bias of rs1058205 (**A**), rs2735839 (**B**), and rs266882 (**C**) under RR vs. WW genetic model

The Egger’s test and Begg’s funnel plot were used to assess the publication bias within the included studies. No obvious evidence of publication bias was indicated in rs1058205 (C-allele vs. T-allele, *t* = −0.13, *P* = 0.909; CC vs. TT, *t* = −0.13, *P* = 0.912; CT vs. TT, *t* = −0.12, *P* = 0.916; CC + CT vs. TT, *t* = −0.12, *P* = 0.916; CC vs. CT + TT, *t* = −0.13, *P* = 0.912), rs2735839 (G-allele vs. A-allele, *t* = 0.57, *P* = 0.626; GG vs. AA, *t* = −0.05, *P* = 0.964; GA vs. AA, *t* = 0.92, *P* = 0.456; GG + GA vs. AA, *t* = 0.47, *P* = 0.682; GG vs. GA + AA, *t* = 0.01, *P* = 0.992), and rs266882 (G-allele vs. A-allele, *t* = −1.57, *P* = 0.361; GG vs. AA, *t* = −1.74, *P* = 0.332; GA vs. AA, *t* = −1.66, *P* = 0.345; GG + GA vs. AA, *t* = −1.71, *P* = 0.336; GG vs. GA + AA, *t* = −1.65, *P* = 0.346). The shape of the funnel plots seemed asymmetrical in the homozygote comparison for the three KLK3 polymorphisms, which means no publication bias was found (Figure 9).

## Discussion

To our knowledge, the pathogenesis and etiology of PCa is still inconclusive. However, the continuous development in molecular biology and immunology methods can provide a basis for the diagnosis of PCa and evaluation of its prognosis [28]. The KLK gene family, consisting of 15 genes spanning in a region of approximately 300 kb on 19q13.4, encodes a cluster of serine-proteases in the human genome [29,30]. KLK3 encodes a PSA, which is mainly expressed in the prostate tissue and is useful in the diagnosis and monitoring of PCa [31,32]. Previous studies have demonstrated the association between KLK3 rs2735839 G allele and higher PSA levels in various ethnicities. Nevertheless, the association between KLK3 polymorphism and PCa susceptibility remains controversial [33,34]. Our meta-analysis primarily aimed to combine previous studies to provide more clear conclusions.

The results of the overall analysis indicated that KLK3 rs1058205 T>C polymorphism was associated with decreased PCa risk, especially in the Caucasians. No association was detected between the KLK3 rs2735839 A>G polymorphism and PCa risk in all comparisons. In addition, no association was observed between rs266882 A>G variant and PCa risk. In population-based studies stratified by source of control, a significant association between the rs1058205 T>C polymorphism and PCa was observed. Furthermore, we assessed the serum expression of KLK3 by ELISA and indicated that individuals who were CC/CT carriers of KLK3 rs1058205 variant had a lower serum expression level than TT carriers. This result shows evidence that mutation from T to C at KLK3 rs1058205 locus may cause lower expression of KLK3. To further demonstrate the influence of KLK3 variants on PCa susceptibility, we used online gene expression mini database to assess the expression of KLK3 in PCa tissues. It indicated that KLK3 expression in PCa tissue was higher than that in normal tissue, which was in line with the results of our study.

Some factors can limit the generalization of the above results. The insufficient number of reported cases on KLK3 variations when specifying different ethnic backgrounds of PCa is one of the main limitations of the present study. Although all eligible studies based on the inclusion criteria were collected, the sample sizes of these studies were not large enough to provide adequate statistical power to assess the association between these polymorphisms and PCa susceptibility in further stratified analysis. The quantitative synthesis of several subgroups may have no sufficient testing power to precisely evaluate the true association. Histological types and Gleason score may also have varied between the evaluated studies. Moreover, the impact of environmental factors such as radiation, toxin, diet, and infectious agent were not evaluated in the analyzed studies. The impact of these factors on susceptibility to PCa should be further evaluated in further experiments. Due to the complex effects of the polygenic nature of complex diseases, including PCa, it is possible that gene-to-gene interactions play a more vital role in an individual’s susceptibility to this disease than single polymorphisms [35]. Furthermore, previous studies showed evidence that manuscript with ‘positive’ results was prone to be accepted faster than that with ‘negative’ findings, which could take longer time to be published in time-lag bias [36].

In spite of these limitations, some key advantages in the present analysis should also be acknowledged. Five case–control studies with 28,830 PCa cases and 28,999 control subjects related to the KLK3 rs1058205 variant, four case–control studies with 5394 cases and 5389 controls concerning the rs2735839 polymorphism, and three case–control studies with 1614 cases and 1981 controls related to rs266882 polymorphism were pooled in our present analysis. Statistical powers of these studies were strongly increased. Furthermore, publication bias was not identified in any of the genetic models, indicating that the results are relatively dependable and stable. We performed stratification analysis and sensitivity analysis to explore the sources of heterogeneity in the present study. Our data indicated that neither ethnicity nor source of controls was the sources of heterogeneity. Sensitivity analysis showed evidence that our data regarding these KLK3 polymorphisms were generalizable and credible.

In summary, our present study showed that rs1058205 polymorphism of KLK3 was a risk factor for PCa development, polymorphism T>C of rs1058205 was associated with decreased susceptibility to PCa particularly in the Caucasian population. Further researches on additional gene–environment interactions are warranted to provide a better understanding on the association between KLK3 polymorphisms and PCa risk.

## Abbreviations

AFR: African
AMR: American
CI: confidence interval
EUR: European
EAS: East Asian
HWE: Hardy–Weinberg equilibrium
KLK: kallikrein
MSMB: microseminoprotein-β
MAF: minor allele frequency
NOS: Newcastle–Ottawa scale
OR: odds ratio
PCa: prostate cancer
PSA: prostate-specific antigen
RFLP: restriction fragment length polymorphism
SAS: South Asian
SLC45A3: solute carrier family 45 member 3
SNP: single nucleotide polymorphism

## References

[1] Siegel R., Ma J., Zou Z. and Jemal A. (2014) Cancer statistics, 2014. CA Cancer J. Clin. 64, 9–29 10.3322/caac.21208 24399786

[2] Ntais C., Polycarpou A. and Tsatsoulis A. (2003) Molecular epidemiology of prostate cancer: androgens and polymorphisms in androgenrelated genes. Eur. J. Endocrinol. 149, 469–477 10.1530/eje.0.1490469 14640986

[3] Chokkalingam A.P., Stanczyk F.Z., Reichardt J.K. and Hsing A.W. (2007) Molecular epidemiology of prostate cancer: hormone-related genetic loci. Front. Biosci. 12, 3436–3460 10.2741/2325 17485312

[4] Duggan D., Zheng S.L., Knowlton M., Benitez D., Dimitrov L., Wiklund F. (2007) Two genome-wide association studies of aggressive prostate cancer implicate putative prostate tumor suppressor gene DAB2IP. J. Natl. Cancer Inst. 99, 1836–1844 10.1093/jnci/djm250 18073375

[5] Chen H., Yu H., Wang J., Zhang Z., Gao Z., Chen Z. (2015) Systematic enrichment analysis of potentially functional regions for 103 prostate cancer risk-associated loci. Prostate 75, 1264–1276 10.1002/pros.23008 26015065

[6] Spisák S., Lawrenson K., Fu Y., Csabai I., Cottman R.T., Seo J.H. (2015) CAUSEL: an epigenome- and genome-editing pipeline for establishing function of noncoding GWAS variants. Nat. Med. 21, 1357–1363 10.1038/nm.3975 26398868 PMC4746056

[7] Paliouras M., Borgono C. and Diamandis E.P. (2007) Human tissue kallikreins: the cancer biomarker family. Cancer Lett. 249, 61–79 10.1016/j.canlet.2006.12.018 17275179

[8] Bansal A., Murray D.K., Wu J.T., Stephenson R.A., Middleton R.G. and Meikle A.W. (2000) Heritability of prostate-specific antigen and relationship with zonal prostate volumes in aging twins. J. Clin. Endocrinol. Metab. 85, 1272–1276 10720075 10.1210/jcem.85.3.6399

[9] Gudmundsson J., Besenbacher S., Sulem P., Gudbjartsson D.F., Olafsson I., Arinbjarnarson S. (2010) Genetic correction of PSA values using sequence variants associated with PSA levels. Sci. Transl. Med. 2, 92 10.1126/scitranslmed.3001513 PMC3564581 21160077

[10] Sun J., Tao S., Gao Y., Peng T., Tan A., Zhang H. (2013) Genome-wide association study identified novel genetic variant on SLC45A3 gene associated with serum levels prostate-specific antigen (PSA) in a Chinese population. Hum. Genet. 132, 423–429 10.1007/s00439-012-1254-3 23269536

[11] Pilia G., Chen W.M., Scuteri A., Orru M., Albai G., Dei M. (2006) Heritability of cardiovascular and personality traits in 6,148 Sardinians. PLos Genet. 2, 132 10.1371/journal.pgen.0020132 PMC1557782 16934002

[12] Lawrence M.G., Lai J. and Clements J.A. (2010) Kallikreins on steroids: structure, function, and hormonal regulation of prostate-specific antigen and the extended kallikrein locus. Endocr. Rev. 31, 407–446 10.1210/er.2009-0034 20103546

[13] Bensen J.T., Xu Z., Smith G.J., Mohler J.L., Fontham E.T. and Taylor J.A. (2013) Genetic polymorphism and prostate cancer aggressiveness: a case-only study of 1,536 GWAS and candidate SNPs in African-Americans and European-Americans. Prostate 73, 11–22 10.1002/pros.22532 22549899 PMC3480543

[14] Zhang L.L., Sun L., Zhu X.Q., Xu Y., Yang K., Yang F. (2014) rs10505474 and rs7837328 at 8q24 cumulatively confer risk of prostate cancer in Northern Han Chinese. Asian Pac. J. Cancer Prev. 15, 3129–3132 10.7314/APJCP.2014.15.7.3129 24815458

[15] Hu J., Qiu Z., Zhang L. and Cui F. (2014) Kallikrein 3 and vitamin D receptor polymorphisms: potentials environmental risk factors for prostate cancer. Diagn. Pathol. 9, 84 10.1186/1746-1596-9-84 24755043 PMC4022449

[16] Cicek M.S., Liu X., Casey G. and Witte J.S. (2005) Role of androgen metabolism genes CYP1B1, PSA/KLK3, and CYP11alpha in prostate cancer risk and aggressiveness. Cancer Epidemiol. Biomarkers Prev. 14, 2173–2177 10.1158/1055-9965.EPI-05-0215 16172228

[17] Lai J., Kedda M.A., Hinze K., Smith R.L., Yaxley J., Spurdle A.B. (2007) PSA/KLK3 AREI promoter polymorphism alters androgen receptor binding and is associated with prostate cancer susceptibility. Carcinogenesis 28, 1032–1039 10.1093/carcin/bgl236 17151093

[18] Eeles R.A., Kote-Jarai Z., Giles G.G., Olama A.A., Guy M., Jugurnauth S.K. (2008) Multiple newly identified loci associated with prostate cancer susceptibility. Nat. Genet. 40, 316–321 10.1038/ng.90 18264097

[19] Wang N.N., Xu Y., Yang K., Wei D., Zhang Y.G., Liu M. (2013) Susceptibility loci associations with prostate cancer risk in northern Chinese men. Asian Pac. J. Cancer Prev. 14, 3075–3078 10.7314/APJCP.2013.14.5.3075 23803082

[20] Penney K.L., Schumacher F.R., Kraft P., Mucci L.A., Sesso H.D., Ma J. (2011) Association of KLK3 (PSA) genetic variants with prostate cancer risk and PSA levels. Carcinogenesis 32, 853–859 10.1093/carcin/bgr050 21421545 PMC3106437

[21] Chen C. and Xin Z. (2017) Single-nucleotide polymorphism rs1058205 of KLK3 is associated with the risk of prostate cancer: A case-control study of Han Chinese men in Northeast China. Medicine (Baltimore). 96, 6280 10.1097/MD.0000000000006280 PMC5348193 28272245

[22] Parikh H., Wang Z., Pettigrew K.A., Jia J., Daugherty S., Yeager M. (2011) Fine mapping the KLK3 locus on chromosome 19q13.33 associated with prostate cancer susceptibility and PSA levels. Hum. Genet. 129, 675–685 10.1007/s00439-011-0953-5 21318478 PMC3092924

[23] Stegeman S., Amankwah E., Klein K., O’Mara T.A., Kim D., Lin H.Y. (2015) A large-scale analysis of genetic variants within putative miRNA binding sites in prostate cancer. Cancer Discov. 5, 368–379 10.1158/2159-8290.CD-14-1057 25691096 PMC4390388

[24] Mantel N. and Haenszel W. (1959) Statistical aspects of the analysis of data from retrospective studies of disease. J. Natl. Cancer Inst. 22, 719–748 13655060

[25] DerSimonian R. and Laird N. (1986) Meta-analysis in clinical trials. Control. Clin. Trials 7, 177–188 10.1016/0197-2456(86)90046-2 3802833

[26] Hayashino Y., Noguchi Y. and Fukui T. (2005) Systematic evaluation and comparison of statistical tests for publication bias. J. Epidemiol. 15, 235–243 10.2188/jea.15.235 16276033 PMC7904376

[27] Wells G.A., Shea B., O’Connell D., Peterson J., Welch V. The Newcastle-Ottawa Scale (NOS) for assessing the quality of nonrandomised studies in meta-analyses. Ottawa Health Research Institute, http://www.ohri.ca/programs/clinical_epidemiology/oxford.asp

[28] Dabir P.D., Ottosen P., Høyer S. and Hamilton-Dutoit S. (2012) Comparative analysis of three- and two-antibody cocktails to AMACR and basal cell markers for the immunohistochemical diagnosis of prostate carcinoma. Diagn. Pathol. 7, 81 10.1186/1746-1596-7-81 22800084 PMC3434074

[29] Yousef G.M., Luo L.Y. and Diamandis E.P. (1999) Identification of novel human kallikrein-like genes on chromosome 19q13.3-q13.4. Anticancer Res. 19, 2843–2852 10652563

[30] Diamandis E.P., Yousef G.M., Luo L.Y., Magklara A. and Obiezu C.V. (2000) The human kallikrein gene family: implications in carcinogenesis. Trends Endocrinol. Metab. 11, 54–60 10.1016/S1043-2760(99)00225-8 10675891

[31] Catalona W.J., Smith D.S., Ratliff T.L. and Basler J.W. (1993) Detection of organ-confined prostate cancer is increased through prostate-specific antigen-based screening. JAMA 270, 948–954 10.1001/jama.1993.03510080052031 7688438

[32] Ilic D., Neuberger M.M., Djulbegovic M. and Dahm P. (2013) Screening for prostate cancer. Cochrane Database Syst. Rev. 1, CD004720 10.1002/14651858.CD004720.pub3 PMC8406915 23440794

[33] Xu J., Isaacs S.D., Sun J., Li G., Wiley K.E., Zhu Y. (2008) Association of prostate cancer risk variants with clinicopathologic characteristics of the disease. Clin. Cancer Res. 14, 5819–5824 10.1158/1078-0432.CCR-08-0934 18794092 PMC2810539

[34] Severi G., Hayes V.M., NeuWng P., Padilla E.J., Tilley W.D., Eggle ton S.A. (2006) Variants in the prostate specific antigen (PSA) gene and prostate cancer risk, survival and circulating PSA. Cancer Epidemiol. Biomarkers Prev. 15, 1142–1147 10.1158/1055-9965.EPI-05-0984 16775173

[35] Wei Q., Cheng L., Amos C.I., Wang L.E., Guo Z., Hong W.K. (2000) Repair of tobacco carcinogen-induced DNA adducts and lung cancer risk: a molecular epidemiologic study. J. Natl. Cancer Inst. 92, 1764–1772 10.1093/jnci/92.21.1764 11058619

[36] Ioannidis J.P. (1998) Effect of the statistical significance of results on the time to completion and publication of randomized efficacy trials. JAMA 279, 281–286 10.1001/jama.279.4.281 9450711

